# Indents

**Published:** 1909-12-15

**Authors:** 


### INDENTS.

☞Brief Articles of Technical or Scientific Value will receive notice and comment. Contributions invited.

*Oral Massage.* Oral massage, with its most effective revolving rubber cup, such as is used in carrying pumice to the teeth, over the gums.—H. E. Latcham, in *Review.*

*Die Metal.* Bismuth 48 parts, cadmium 13 parts, lead 19 parts, tin 20 parts. This easily fusible metal can be poured into wet plaster.—*Zeitschrift fur Zahnarztliche Orthopadie.*

*Odorless Deodorant for Foul Toilets, Cuspidors, etc.*

| | |
|---|---|
| Ferric chlorid | 4 parts |
| Zinc chlorid | 5 parts |
| Aluminum chlorid | 5 parts |
| Calcium chlorid | 4 parts |
| Magnesium chlorid | 3 parts |
| Water to make | 100 parts |

Add to each gallon 10 grains thymol and ¾ oz. oil of rose.

Dr. Trueman in *Brief.*

*How to protect Gold Work from Amalgamation.* Before inserting an amalgam filling in a mouth containing gold, especially to the amalgam is in close proximity if a gold filling or crown, dry the gold work and varnish it with a coat of sandarac varnish.—A. F. Donahower, Philadelphia.

*Eastern Indiana Dental Association.* The Eastern Indiana Dental Association, after the selection of Cambridge City as the next meeting place, elected officers as follows: Dr. Charles Niese, of Cambridge City, president; Dr. C. C. Miller, of Plainfield, vice-president, and Dr. O. A. Martin, of Richmond, secretary-treasurer.—*Summary.*

*Stopping Pain.* In Chicago, at a recent clinic, Dr. Keefe demonstrated that any pain arising from the fifth nerve could be temporarily stopped by making two or three injections of equal parts of water and alcohol into the nostrils by means of a watch-case atomizer. The pain would disappear in from ten to fifteen seconds.—Dr. W. E. Tennant, *Review.*

*Anesthetizing the Pulp.* For anesthetizing pulps use a solution of cocain mixed with glycerine to a thick plastic consistence. One or two drops of adrenalin chlorid added will prevent hemorrhage. Glycerine is an effective menstruum for cocain, and owing to its grea affinity for water is easily absorbed by the pulp itssue.—*Deutsche Zahnarztliche Zeitung.*

*Argyrol.* I do not think there is any agent that is more potent in the destruction of pathogenic microorganisms we find in these surfaces than argyrol. It has been shown conclusively that argyrol will penetrate as deeply, if not more deeply, into the tissues, and destroy microorganisms more certainly than most any of the agents with which we are acquainted.—Dr. T. W. Brophy in *Review.*

*Dentists for State Institutions.* Governor Gillett, of California, has signed his state dental surgeon bill. This bill provides for the appointment of a state dental surgeon at a salary of $200 per month. The dentist is to put in his time traveling among the state insane institutions, of which there are five, and attending to the dental needs of the inmates.—*Summary.*

*Flies, to Destroy.*

| | |
|---|---|
| Formaldehyde solution | 3 parts |
| Milk - - - - | 4 " |
| Water - - - - | 13 " |

Mix, and pour in shallow plates or trays accessible to the flies. They are fond of it, and quickly fall victims to the tipple. They are killed, not by the fumes of the formaldehyde, but by drinking it.—*Merk's Report, June*, 1909.

*Drainage of Abscesses.* In all cases of abscesses in the mucous tissues of the mouth I insist on the use of 95 per cent. carbolic acid on the tent. A very small amount retained in the gauze or cotton is sufficient to cauterize the lips of the incision, making the opening freer, promoting the egress of pus and preventing the ingress of new infective material.—G. V. Black in *Northwestern Dental Journal.*

*Banded Crowns.* To me a crown made without a band flavored of careless dentistry until recently, but as I go deeper into pyorrhea and prophylactic work I see less use for a banded crown, for in a majority of cases there is always more or less irritation until the gum tissue has receded to the top of the band. It is impossible, in a majority of cases to properly treat, for pyorrhea, a root which wears a banded crown.—Frank H. Skinner, in *Review.*

*Heating the Investment Before Casting* It is not necessary to have the flask exceedingly hot while casting, except there should be present in the flask porcelain teeth or pieces of gold or platinum that are intended to be picked up by the casting. The presence of the teeth requires a high heat to prevent checking, and with gold or platinum in the mold a high heat is needed to ensure a homogeneous union.—Dr. J. G. Lane in Philadelphia *Dental Brief.*

*A Medical as well as a Dental Course to be Required.* A step toward the requirement of a four years' course in medicine, in addition to a special course in dentistry, was taken by the North Carolina Dental Society when a resolution to that effect was adopted by the society in convention at Asheville, N. C., June 24. The resolution, however, has as yet no binding force on the legislature, and it is not proposed to make it effective until 1915.—*Spartansburg Herald.*

*Inter-State Reciprocity.* Reciprocal exchange of license has been arranged between Ohio, and Michigan, Indiana, Iowa, New Jersey and the District of Columbia. An Ohio dentist in legal and reputable practice for five years may practice, under the agreements, in the other states without examination, while dentists from those states can enter Ohio under the same conditions. Dr. F. R. Chapman, of Columbus, is secretary of the Ohio board. —*Columbus, Ohio, Journal.*

*Dissolving Cement from Crowns.* If a Davis crown pin should break from the root, the pin may be easily removed from the porcelain by boiling in equal parts of sulphuric acid and water. In a few minutes the cement will be entirely dissolved and thepin will drop out.

This is also good for dissolving cement from shell crowns that are to be remounted.

A piece of cement the size of a pea will completely dissolve in about two minutes.—Dr. W. L. Reed, Mexico. Mo,

*Examination of School Children.* In his annual report, the medical officer of health for Yarmouth sets forth the results obtained by the weighing, measuring, and physical examination of the children in the public schools. He strongly urges that it would be a great gain if girls up to the age of ten had their hair kept short. Of boys, 971 were examined and 899 girls, in school, and carious teeth were found in 857 cases, showing the high proportion of 46 per cent. One hundred and forty-seven children were found with defective vision, of whom 18 per cent. were already provided with glasses, mostly obtained through Yarmouth Hospital.

*New Examination for Dentists in Germany.* A new examination for dentists has been provided by the German federal council. This gives the right of conferring license on dentists to the authorities of each State of the empire which has one or more national universities. The production of a certificate of graduation is a condition for entrance on the study. The time spent in military duty cannot be reckoned in the prescribed period of study. The new regulations take effect on October 1 of this year.—*Journal* (Berlin Letter).

*Drying Root Canals.* I have been somewhat surprised to find that a glass tube drawn out to about the size and shape of a lingual root of an upper molar tooth and filled with alcohol and its apex sealed by placing into a little wax, cannot be dried with as many as fifty blasts of hot air with the ordinary chip blower; water required one hundred blasts to reduce it perceptibly. A hot metal root instrument will not dry it readily. Nothing but thorough absorbing, as by means of cotton twisted upon a broach.—E. B. Lodge.

*Oral Hygiene for the Public.* But why talk? Why not do something? The only way to educate the public is for the profession to take steps to deliver lectures before teachers, institutes, public and private schools, to medical students and medical societies and the classes of training schools for nurses. By so doing the public will eventually become educated to the great importance of this vital subject. Literature of this subject, carefully prepared, under the auspices of local, state, or the National Dental societies, should also be furnished the press for publication.—Dr. B. L. Thorpe, in *Dental Summary.*

*Cast Gold Inlay* A gold inlay is the "greatest ever" for a badly broken down bicuspid tooth. I make them and bless the man who conceived the idea. But that all or any considerable number of cavities should be inlayed is impossible to my conception of correct practice. When cavities are treated at the proper time, with correct cavity preparation and filling, inlays are certainly not indicated. The man who inlays the majority of his cavities must be lacking in the skill to insert gold fillings and amalgam fillings, or else his commercial instincts are greater than his professional ones.—S. H. Voyles in *Brief*.

*To Soften Moldine Which Has Become Hard.* It often happens that from frequent use, or long exposure to air, moldine loses its placidity. The usual method of adding glycerine is more or less unsatisfactory, even when all instructions are carefully followed. The best process to use is to place the moldine to be softened in a vessel, to which a sufficient quantity of water, to cover the moldine is added. One or two spoonfuls of glycerine are added to the water. The moldine is then heated until the water has evaporated after which the moldine may be found having its original placidity.—Le Laboratoire.

*Pulp Protection.* Prior to filling a deep occlusal or other cavity which closely approaches the pulp, it is a wise precaution to seal in the cavity an anodyne and antiseptic dressing, such for instance as the menthol-thymol-phenol preparation suggested by Dr. Buckley, allowing it to remain about a week; then place a liberal amount of copper phosphate cement in the deeper portion of the cavity to serve as a foundation for the permanent filling. This protects the pulp from thermal changes, and from pressure during the introduction of the filing.—Dr. F. T. Weeks in *Brief*.

*Why the Pulp Canal is so Difficult to Reach.* Dr. G. V. Black suggests that much of thoughtless burring away of the floor of the pulp cavity. Each pulp canal opens into the pulp cavity by a funnel-shaped opening, forming an unerring guide for the broach, and suggestive to the close observer as to their number and location. If these are burred away these land-marks are destroyed, and, furthermore, the openings, with nothing to guide the instrument to them, are then located in positions difficult of access, especially when introducing the filling, as the filling material seriously obstructs the view.—*Dental Brief.*

*The Teeth of Cleveland School Children to be cared For* In a letter submitted to the Board of Education of Cleveland, Ohio, three dental societies volunteered to establish free dental clinics for school children. Their proposition was accepted. Dentists in charge of the clinics will work without remuneration. They will repair the children's teeth, keep them in good condition and teach the child how to properly care for them. The decision on the part of the dentists followed a statement by Health Officer Friedrich that seventy percent. of the school children had decayed teeth. Superintendent Elson will decide during the summer upon the four schools where clinics will be opened.—*Cleveland News.*

*Investment for Casting Gold.* After a series of carefully conducted experiments I found asbestos was not a desirable ingredient in an investment for gold casting. There seemed to be a gas arising from the heated asbestos that so interfered with the introduction of the gold that round corners were obtained. Pumice stone was not suitable because an investment containing it lacked strength, and also seemed to be porous or granular, resulting in a rough surfaced casting. Finely powdered firebrick was uncertain. Results were good, bad, and indifferent; why I was unable to determine. Chalk contracted under heat. Of course, plaster of Paris was present in varying quantities in all of these trials. Plaster one, and silex three by weight, has proved excellent; there is no contraction, and no pickling is needed to clean the casting made in it.—Dr. J. G. Lane, Philadelphia.

*A Systemic Vice.* I still think, when we come down to the last analysis of the question, that we will be in agreement upon the point that this question of the defensive forces of the body in its relation to bacteriological infection is a factor which, when properly expressed, in terms that are understandable to all men, will mean that which I have tried to express when I say that the lack of it constitutes a systemic vice. There is a peculiar constitutional relationship to the pyorrhea invasion question quite apart from the local manifestations of it that we will have to take into account and to combat along the lines of which the essayist has spoken if we are really to cure the disease. —Dr. E. C. Kirk in *Western Journal.*

*Aluminum to give a Satin Finish.* There are two methods in use to give aluminum a satin finish, the chemical and the mechanical. The chemical: First remove the grease by dipping in benzine, then dip in a solution of caustic soda or caustic potassa (potassium or sodium hydrate) strong enough to blacken the metal. Remove the article from this bath and dip in a mixture of acids, one part sulphuric and two parts nitric, and finally in a mixture of equal parts vinegar and water, and dry in hot sawdust. The mechanical method is by the use of a sand blast machine, the sand used being very fine. By holding the aluminum article in the stream of fast moving sand a lasting and very beautiful mat surface is produced.—*Work.*

*Dentists and Doctors to Get Together.* A movement has been set on foot to bring the professional men of Dayton, Ohio, into closer relationship. The arrangement calls for joint meetings of men whose professions are closely related and if the plans are carried out as intended, in the future, doctors and druggists will at times have joint meetings of their respective associations. Dentists and doctors will also meet together at times.

Recently the Miami Valley Dental Society and physicians met together at the Beckel, when an address on prenatal influence with relation to the teeth and dental operations was discussed by Dr. T. A. McCann—*Dayton Herald.*

*A Safe Method of Adapting Wax Inlay Models.* When the cavity is filled with wax, stretch a piece of thin rubber dam over it and draw tightly. Both ends of the rubber can usually be held by the fingers of one hand in such a way that the tension will force the wax into the cavity. Burnish through the rubber till all margins are adapted and all but the finest carving of the wax has been done.

In approximal models, separation must be made between the wax and the adjoining tooth, preferably with a thin ribbon-saw. If it is necessary to build additional contours, add beeswax to the finished model.—E. S. Ulsaver, *Dental Digest.*

*Origin of Dentistry.* The Ebers papyrus, the oldest medical manuscript known, treats of diseases of the teeth and gums, and in this interesting book of ancient Egypt, dating as far back as 3700 before Christ, no less than fourteen prescriptions for the treatment of several dental diseases may be found. The initiator of dental surgery undoubtedly the Greek Aesculapius, who flourished thirteen centuries before the Christian era, and according to traditions invented several important curative methods. In fact, Cicero says he invented the probe and was the first to skillfully bind up a wound, but it is particularly interesting to us to hear that he was the originator of the operation of the extraction of teeth. It may be that he was the inventor of the odontagogon, which long years after, we are told, was preserved in the temple of Apollo at Delphi. That this ancient forceps was made of lead may serve as a hint that no great force was to be used in the removal of the teeth.—Chas. McManus in *Dental Review.*

*Hard Work of a Dentist.* A good dentist gives the very blood of his veins to his patients for there is no professional work harder on body and brain than dentistry. Some of the best dentists seem crossest. Sit down, open your mouth and let the dentist do his work right and don't grab his hands or try to tell him how, because this bothers him and makes his work fail. A workingman gets all confused if somebody constantly grabs his tools and meddles with him, and anybody except a dentist would quit the job in disgust. Some people expect too much of dentists, expect an old decayed tooth filled from the enamel to the end of the root never to decay again, to last for life; expect it to be better than a tooth never decayed or filled.—*New York Press.*

*Weighted Lower Dentures.* On the lower jaw I used to recommend in a good many cases the Watt's metal plates. The second plate I used for myself was one of that kind. After wearing the plate for a short time I went into the laboratory and leaned over to speak to a student, when the plate slipped forward, owing to its excessive weight, there being no ridge to hold it. That night as soon as I lay down, the plate slipped into the cheek. If the patient has a fair ridge, that trouble will not occur. From experience, however, I have learned something more: On the lower jaw a light rubber plate can be managed as well as in all respects a heavy plate. Weight is not needed. Patients often complain of a heavy plate on the lower jaw.—Dr. Haskell in *Cosmos.*

*Construction of Crowns.* A study of the forms of the teeth shows in most cases a construction of the gingival enamel margin, thus producing a recess which affords some protection to the gingivae against the excursions of food. This form of development is quite marked in the cingulae of the incisors where the gingival border is exposed to constant action from the incision of food. In constructing crowns, if this form be given them, greater protection will be afforded the gingivae and peridental membrane, than if the sides of the crown are parallel. By restoring proximal contact the tissues in the interproximal spaces are protected, while correct occlusion obviates undue stress being brought upon the peridental membrane in mastication.—J. H. Prothero, in *Review*.

*Iodoformogen, to Replace Iodoform.* Iodoformogen is prepared by precipitating a solution of albumin with alcoholic solution of iodoform and heating the precipitate to 148 deg. F. It is a very fine, voluminous, light yellow, nearly odorless powder, non-hygroscopic and non-conglutinating. It is insoluble in water and can be sterilized at 212 deg. F. without decomposition or change. It has all the desirable properties of iodoform without its unpleasant odor, with the further advantage that as its contents of iodoform is very slowly given off when the drug comes in contact with the wound-surfaces the action of iodoformogen is more prolonged than that of iodoform itself. It is non-irritating. In dental practice it may be used precisely as is iodoform.—Dr. J. M. Woodle in *Dental Cosmos*.

*How to teach a child to masticate food properly.* The first thing to do in giving instructions is not to teach anything wrong. Nine-tenths of the children, especially in their infancy, are taught that they must not leave anything on their plate, and also that they must eat when mamma eats whether they are hungry or not. If the child is watched and guarded against misinformation, he will begin to pick out for himself what is the right thing to eat. The great affliction that we suffer from this age is the plethora of food by which we are surrounded, and the continuous suggestion to eat, even to the point of gluttony. That, in connection with what I call aggressive hospitality—*i. e.* hosts wanting to stuff us to see the effect of the food—is the condition by which we are surrounded all the time, and this misconception forced on the minds of the children is generally detrimental to them.—Mr. Fletcher in *Cosmos.*

*A "Queer" Dyspepsia Cure.* A much advertised dyspepsia cure yielded on analysis the following:

| | |
|---|---|
| Coarse quartz sand | 87.5 |
| Soluble matter | 12.5 |

The soluble matter was found to be rock candy and syrup. Directions: Take a scant teaspoonful after a light meal, and two or three after a heavy one. Do not swallow it at once, but grind it around between the teeth until well saturated with saliva.—*Journal A. M. A.*

It might be a help by keeping the grinders sharp, and so assisting mastication (?). Thrift would suggest, however, buying the sand by the bushel rather than pay three dollars for a small package. Nostrum analysis reveals some queer mixtures "warranted pure," as was this. (Pure humbug.)—*Brief.*

*An Adhesive Cement.* The following cement will stick to anything.

| | |
|---|---|
| Clean gum arabic | 2 oz. |
| Fine starch | 1½ oz. |
| White sugar | ½ oz: |

Pulverize the gum arabic and dissolve it in as much water as a laundress would use for the quantity of starch indicated. Dissolve the starch and sugar in the gum arabic solution. Cook the mixture in a vessel suspended in boiling water until it becomes clear. It should be as thick as tar, and should be kept so. To keep from spoiling, put in a small lump of camphor, or a few drops of oil of cloves or sassafras. This cement is very strong and will stick perfectly to glazed surfaces, and is an ideal glue for repairing broken rocks, minerals, or fossils.—E. Viall, in March, 1909, *Electrician and Mechanic.*

*Dressing for Putrescent Canals.*

Following are two prescriptions, one a powder and the other a liquid, mixed like a cement, very thin, and makes an ideal dressing in putrescent canals as a first application, or mixed very stiff makes a good permanent filling in abscessed root canals. Many abscessed teeth can be cured permanently with it:

| | |
|---|---|
| LIQUID: | |
| Oil of Cloves | ʒj |
| Creosote | ʒj |
| Formalin | gtts. ij |
| POWDER: | |
| Thymol | grs. xxx. |
| Alum | grs. xx. |
| Zinc Oxid | grs. xx. |
| Morphine Sulphate | grs. x. |

A. M. Schoenbrod in *Review*.

*Constructing Abutments for Replacing Lateral.*

The semi-jacket is admirably suited to cuspid abutments, and by adopting the casting method is comparatively simple in construction. The cuspid is the most favorable for this form of attachment and in many instances will serve alone to support a lateral dummy.

Where the bite is not too close and the patient will submit to the removal of a portion of the lingual surface of enamel, and drilling two or three small pin holes into the dentin between the pulp and periphery of the tooth, a very satisfactory piece of work may be done in this way. This method will necessitate anchoring to both cuspid and central and requires a high degree of skill in successful application.—F. E. Roach, Chicago.

*Silicious Cements.*

I have inserted enamel fillings with ivory, bone, tortoise shell, agate, celluloid and glass instruments; used a special glass slab, mixed by the old and by the new methods; used vaseline and didn't use vaseline; in short, I have fought gloriously and have been stung.

Many of my porcelain inlays were garish and even impudent in their desire and ability to be conspicuous, but my enamel fillings are wonderfully shrinking and retiring in disposition. Some of them have retired via the alimentary canal, others have cloaked their shrinking timidness in a garb of black. The obsequies were attended by a mournful patient and your apologetic essayist.

In my hands the silicate cements are as certainly uncertain as the old-fashioned tin amalgams, and more deadly in effect. And yet what a temptation they are! I frequently think it must be a dream, or try to think that it must be all my own fault that so many enamel fillings go bad.—S. H. Voyles in *Brief*.

*Silicate Cements.* The silicate cements seem to resist the action of the fluids of the oral cavity better than their predecessors, but time has shown that they are not insoluble and that they do slowly wash away on their exposed surfaces. For this reason they cannot be regarded as permanent fillings, and patients should not be allowed to consider them as such. Their peculiarly natural appearance makes them very attractive and desirable, but they should not be permitted to supersede porcelain inlays, for they possess neither the beauty nor the permanence of porcelain.

When it is desired to insert a filling that shall be next to porcelain in naturalness, and is intended to serve its purpose for a time, the silicate cement is indicated. In the anterior teeth of the deciduous set, or in the teeth of adults where the tooth-structure is of such a character as not to be able to resist the influence of caries, silicate cements find their field of greatest usefulness. In cases also where large restorations are demanded in the anterior teeth, on surfaces removed from occlusal strain, and when porcelain inlays are beyond the means of the patient, the silicates can be employed to great advantage.

Teeth greatly weakened by caries, where it may be desired to avoid the more elaborate operation of crowning as long as possible, may be made presentable and even strengthened by silicate fillings. While, as has been said, fillings of this type cannot be classed as permanent, their life may be greatly extended by giving attention to certain details of procedure. Among these are the proper shaping of the cavity; its protection from moisture both before and after the introduction of the material; thorough mixing of the cement and its prompt though careful placement, and finally after the surface has been made to conform to the contour of the tooth, the flowing of melted paraffin over its surface. If after applying the paraffin it is good over with a hot burnisher several times, the cement will absorb sufficient of the melted material to render it impervious to moisture, and at the same time the translucence and life-like appearance of the filling will be increased.

Like every other filling material, the silicate cement has its appropriate place. Used with discretion and with due regard for the existing conditions, there is no reason why it should not find a permanent place among the list of tooth-preservatives. The great danger lies in the probability of its being employed too frequently and in cases where other and more permanent materials would serve a better purpose. —S. H. Guilford, *Garretsonian.*

